# Association between the length of stay in rehabilitation and mortality among the adults with Parkinson’s disease: 2009–2019 Korean National Health Insurance Service Databases

**DOI:** 10.3389/fnagi.2024.1428972

**Published:** 2024-08-05

**Authors:** Suyeong Bae, Ickpyo Hong, Min Seok Baek

**Affiliations:** ^1^Department of Occupational Therapy, Graduate School, Yonsei University, Wonju, Gangwon-do, Republic of Korea; ^2^Department of Occupational Therapy, College of Software and Digital Healthcare Convergence, Yonsei University, Wonju, Gangwon-do, Republic of Korea; ^3^Department of Neurology, Wonju Severance Christian Hospital, Yonsei University Wonju College of Medicine, Wonju, Gangwon-do, Republic of Korea

**Keywords:** rehabilitation, mortality, Parkinson’s disease, longitudinal studies, occupational therapy, physical therapy

## Abstract

**Background:**

Rehabilitation is recognized as an effective means of alleviating the symptoms of Parkinson’s disease (PD) and improving the physical and cognitive functions of patients with PD. However, research often focuses on short-term outcomes such as functioning and quality of life. This study investigated the association between the length of stay in rehabilitation and mortality among patients with PD.

**Methods:**

Using the Korean National Health Insurance Service database, we identified 636 participants diagnosed with PD who received rehabilitation. The main outcome was all-cause mortality. We used a Cox proportional hazards regression model to examine the relationship between length of stay in rehabilitation and mortality among patients with PD.

**Results:**

The final sample comprised 374 females (58.81%) and 262 males (41.19%). A survival analysis revealed a significant association between the length of stay in rehabilitation and mortality, with a decrease in mortality of 16.1% in patients with PD who received one year of rehabilitation (hazard ratio = 0.839, 95% confidence interval = 0.788–0.895).

**Conclusion:**

Our findings underscore the potential benefits of timely implementation of rehabilitative interventions in patients with PD and the need for comprehensive and long-term rehabilitation strategies. It also highlights the necessity of such services for patients with PD and the importance of developing patient-centered rehabilitation guidelines.

## 1 Introduction

Parkinson’s disease (PD) is a degenerative central nervous system disorder caused by the death of dopamine neurons. In recent years, there has been a global increase in the incidence of PD. At present, the prevalence of PD is reported to be 315 people per 100,000 worldwide (Han et al., 2019). A study using national health insurance claims data reported an average PD prevalence in Korea between 2012 and 2015 of 171 per 100,000 (Han et al., 2019). According to the big data open portal of the Health Insurance Review and Assessment Service, 96,499 people were treated for PD in Korea in 2016. In 2020, this figure rose to 111,311, an increase of 14,812 people. The annual average growth rate in PD diagnoses is 3.6% (Health Insurance Review & Assessment Service, 2023).

Previous studies have reported that rehabilitation can be used as a non-pharmaceutical means of increasing function in patients with PD (Borrione et al., 2014; Seo et al., 2018). The Act on Medical Technologists of Korea stipulates that the medical service technologists permitted to provide rehabilitation services are limited to physical therapists and occupational therapists (Republic of Korea, 2020). Rehabilitation services aim to improve independence, mental and physical function, and physical fitness (Borrione et al., 2014). Physical activities and cognitive therapy are used as intervention tools in rehabilitation (Bouça-Machado et al., 2020).

Rehabilitation services have been shown to effectively reduce both the physical and cognitive symptoms of PD (Welsby et al., 2019; Doucet et al., 2021; Elena et al., 2021; Sanchez-Luengos et al., 2021; El Hayek et al., 2023). Rehabilitation interventions for PD are designed to promote functional independence and increase participation in daily life, including gross and fine movements, and activities of daily living (Welsby et al., 2019; Sanchez-Luengos et al., 2021; El Hayek et al., 2023). A systematic review has reported that physical therapies such as task-related trunk training and vestibular rehabilitation improve the physical functioning of patients with PD. These approaches are also effective non-pharmaceutical methods of enhancing mobility (El Hayek et al., 2023). Another systematic review found that home-based occupational therapy improves the upper limb function of patients with PD and increases their ability to perform occupational tasks (Welsby et al., 2019).

As mentioned above, while studies have demonstrated that rehabilitation services effectively improve the functional status of patients with PD, they have not established an association with long-term outcomes such as mortality. This oversight can be attributed to two factors: first, previous studies on the efficacy of rehabilitation services have predominantly focused on immediate functional improvements post-intervention (Tomlinson et al., 2012; Sanchez-Luengos et al., 2021; El Hayek et al., 2023); second, those studies that have included follow-up data followed-up for only short periods or had small sample sizes (Tomlinson et al., 2012). There has been a lack to investigate the long-term effects of rehabilitation services on the functional status of patient with PD through sustained observational follow-ups (El Hayek et al., 2023). Although some studies have shown that physical activity is associated with reduced mortality, they have not addressed the association of structured rehabilitation services with long-term outcomes such as mortality in patients with PD (Yoon et al., 2021; Zhang et al., 2022). Thus, investigating the relationship between rehabilitation and mortality is necessary to determine the need and value of rehabilitation services for this patient population. Our study addresses these research gaps using longitudinal national health insurance data to examine the association between rehabilitation and mortality in PD over an extended follow-up period. This offers greater insight into the long-term benefits of targeted rehabilitation services.

## 2 Materials and methods

### 2.1 Data source and participants

The study data were the 2009–2019 cohort samples of the Korean National Health Insurance Service (NHIS). The NHIS provides anonymized, standardized datasets covering insurance premiums, health examination results, medical treatment details, and long-term care insurance data. The datasets we searched in this study consisted of sample cohort datasets, health examination cohort datasets, and older adult cohort datasets. The NIHS database includes the month of death, cause of death, medical illness details, diagnosis, pharmaceutical treatment, and demographic characteristics of patients. These are summarized in several tables (Kim et al., 2022).

Participants in this study had been diagnosed with PD and had the G20 code [International Classification of Diseases-10th Revision (ICD-10)] and V124 code in the claims databases. V-codes are defined by the Registration Program for Rare Intractable Diseases of Korea and are used for co-payment claims related to rare and incurable conditions, as well as severe diseases such as cancer (Park et al., 2019; Kim et al., 2021; Yoon et al., 2021). PD is among the degenerative disorders supported by the co-payment policy in Korea (Koo et al., 2023). The study’s exclusion criteria were (1) Individuals diagnosed with PD before 2008; (2) Those who had never received rehabilitation services; (3) Individuals with missing data on the study variables. The data used in this study was approved by the NHIS Inquiry Commission and the Institutional Review Board (IRB) of Wonju Severance Christian Hospital (IRB no. CR321308). The study was also reviewed and approved by the IRB of the Yonsei University Mirae Campus (IRB no. 1041849-202307-SB-128-01).

### 2.2 Study variables

The main outcome was all-cause mortality between January 1, 2009 and December 31, 2019. The primary independent variable was the length of stay in rehabilitation. Study participants identified by claims codes (MM105, MM301, MM302, MM111, MM112, MM113, and MM114) following a diagnosis of PD were considered for receipt of physical and occupational therapy rehabilitation services. A more detailed description of the rehabilitation codes is provided in Supplementary Table 1. The independent variable was the length of stay in rehabilitation. This was calculated from the first year of rehabilitation to the year of death year for those who died, and from the first year of rehabilitation to December 2019 for those who survived.

The covariates were sex, age at PD diagnosis, Charlson Comorbidity Index (CCI) score, total time in receipt of rehabilitation services, levodopa equivalent daily dose (LEDD), health insurance type, region, and insurance premium deciles. The CCI consists of 13 chronic diseases, including myocardial infarction, congestive heart failure, peripheral vascular disease, cerebrovascular disease, chronic pulmonary disease, rheumatologic disease, peptic ulcer disease, diabetes without chronic complications, diabetes with chronic complications, hemiplegia or paraplegia, renal disease, moderate or severe liver disease, and metastatic solid tumors (Hude et al., 2005; Kim, 2016). These 13 chronic diseases are organized into three categories (0, 1, or more than 2). The total time in receipt of rehabilitation services was calculated by multiplying the total number of times rehabilitation services were received, from the point of diagnosis to the end of the observation period, incorporating the treatment duration for each rehabilitation service code. The LEDD was calculated as the prescribed LEDD at the time closest to the dates one year before and after the participants first received rehabilitation services (Tomlinson et al., 2010). The distributions for the total time in receipt of rehabilitation services and LEDD among the study participants were highly skewed so log-transformed total time in receipt of rehabilitation services and LEDD were used for data analysis.

### 2.3 Statistical analysis

Continuous variables were summarized using means and standard deviations, and categorical variables were summarized using frequencies and percentiles. The Cox proportional hazards regression method was used to examine the association between the length of stay in rehabilitation and mortality, with the point estimates presented with hazard ratios (HR) and 95% confidence intervals (CI). For sensitivity analysis, we examined the association between the length of stay in rehabilitation and mortality after dividing the sample into those who had received only occupational therapy, those who had received only physical therapy, and those who had received both occupational and physical therapy. Data management and analysis were performed using SAS version 8.39 software (SAS Institute Inc., Cary, NC).

## 3 Results

### 3.1 Participant selection

**FIGURE 1:**
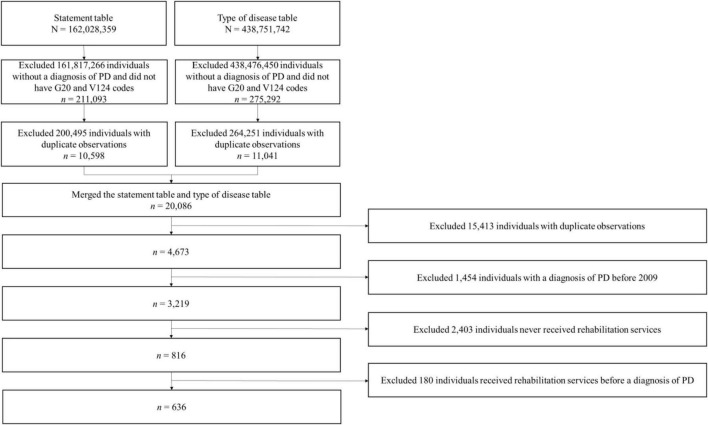
Flow diagram of study cohort selection procedures. The statement table includes the start date of medical care, the principal diagnosis, any secondary diagnosis, surgery status, days of care, and days of outpatient visit or admission. The type of disease table includes the start date of medical care, the medical care exceptional disease symbol, the detailed specialty subject code, and the disease classification code.

Figure 1 presents a flow diagram of the study cohort selection procedures and an explanation of the data tables from the database that were used for data collection. We extracted our data from the statement table and the type of disease table (*n* = 162,028,359, *n* = 438,751,742, respectively). We then removed the data of participants without PD (*n* = 161,817,266, *n* = 438,476,450, respectively), and duplicated data (*n* = 161,817,266; *n* = 438,476,450, respectively) from the two tables. The final sample comprised 636 patients with PD.

### 3.2 Demographic and clinical characteristics of study participants

Table 1 presents the demographic characteristics of the included patients with PD. There were 374 females (58.81%) and 262 males (41.19%), and their mean age at PD diagnosis was 72.17 [standard deviation (SD) = 9.16]. Among these, 278 (43.71%) patients died during the study follow-up period between 2009 and 2019. There were 313 (49.21%) patients with CCI scores higher than two. The mean time in receipt of rehabilitation services was 1329.01 min (SD = 3408.70 min).

**TABLE 1:** Demographic characteristics of study participants (*N* = 636).

Variables	*N* (%)
Age at PD diagnosis, mean (SD)	72.17 (9.16)
**Sex**	
Female	374 (58.81)
Male	262 (41.19)
**Charlson Comorbidity Index**	
0	195 (30.66)
1	128 (20.13)
> 2	313 (49.21)
**Health insurance type**	
Regional subscriber	163 (25.63)
Workplace subscriber	430 (67.61)
Medical aid beneficiary	43 (6.76)
**Region**	
Capital region	267 (41.98)
Non-capital region	369 (58.02)
**Insurance premium deciles**	
0–2	108 (16.98)
3–8	250 (39.31)
9–10	278 (43.71)
LEDD*, mean (SD)	5.79 (2.00)
Frequency of OT receipt, mean (SD)	17.75 (61.40)
Frequency of PT receipt, mean (SD)	33.25 (77.81)
Frequency of rehabilitation services receipt, mean (SD)	51.00 (130.60)
Total time receiving OT (min, mean (SD))	331.368 (1316.45)
Total time receiving PT (min, mean (SD))	997.64 (2334.25)
Total time receiving rehabilitation services (min, mean (SD))	1329.01 (3408.70)

CI, confidence interval; OT, occupational therapy; PT, physical therapy; SD, standard deviation. *LEDD was log-transformed due to a non-normal distribution.

### 3.3 Association between all-cause mortality and length of stay in rehabilitation

Table 2 presents the hazard ratios of all-cause mortality according to the length of stay in rehabilitation. There was a significant association between the length of stay (years) in rehabilitation and mortality (HR = 0.839, 95% CI = 0.788 to 0.895, *p* < 0.0001). Among the covariates, age at PD diagnosis and sex were significantly associated with mortality (HR = 1.068, 95% CI = 1.049 to 1.088, *p* < 0.0001; HR = 1.802, 95% CI = 1.410 to 2.304, *p* < 0.0001, respectively). However, neither the total time in receipt of rehabilitation services, health insurance type, region, insurance premium deciles, nor LEDD was associated with mortality in patients with PD.

**TABLE 2:** Mortality hazard ratios according to the length of stay in rehabilitation in patients with Parkinson’s disease (*N* = 636).

Variables	Estimate	Hazard ratio (95% CI)	*p*-value
Length of stay in rehabilitation (year)	–0.175	0.839 (0.788 to 0.895)	<0.0001*
Age at PD diagnosis	0.066	1.068 (1.049 to 1.088)	<0.0001*
**Sex**			
Female	Ref.		
Male	0.589	1.802 (1.410 to 2.304)	<0.0001*
**Charlson Comorbidity Index**			
0	Ref.		
1	0.844	2.326 (1.596 to 3.392)	<0.0001*
> 2	0.933	2.543 (1.837 to 3.519)	<0.0001*
**Health insurance type**			
Regional subscriber	Ref.		
Workplace subscriber	0.275	1.316 (0.983 to 1.762)	0.0651
Medical aid beneficiary	0.388	1.474 (0.778 to 2.794)	0.2341
**Region**			
Capital region	Ref.		
Non-capital region	–0.143	0.867 (0.678 to 1.108)	0.2536
**Insurance premium deciles**			
0∼∼2	Ref.		
3∼8	0.004	1.004 (0.638 to 1.581)	0.9858
9∼10	–0.136	0.873 (0.555 to 1.373)	0.5562
LEDD**	0.017	1.018 (0.958 to 1.081)	0.5735
Total time in receipt of rehabilitation services**	0.009	1.009 (0.935 to 1.088)	0.8258

CI, confidence interval. *p < 0.0001 **LEDD and total time were log-transformed due to non-normal distributions.

### 3.4 Association between all-cause mortality and length of stay in rehabilitation according to the type of rehabilitation services received

Table 3 presents the hazard ratios for mortality associated with the duration of rehabilitation according to the type of rehabilitation services received. For individuals who received only occupational therapy, there was no significant association between any of the variables and all-cause mortality, except region. However, a significant association was observed between the length of stay in rehabilitation and all-cause mortality for those who received only physical therapy, as well as for those who received both occupational therapy and physical therapy, with HRs of 0.835 (95% CI = 0.733 to 0.951, *p* = 0.0068) and 0.842 (95% CI = 0.777 to 0.912, *p* < 0.0001), respectively.

**TABLE 3:** Mortality hazard ratios according to the length of stay in rehabilitation and type of rehabilitation services received in patients with Parkinson’s disease.

Variables	Only OT (*n* = 39)		Only PT (*n* = 180)		Both OT and PT (*n* = 417)	
	Estimate	Hazard ratio (95% CI)	Estimate	Hazard ratio (95% CI)	Estimate	Hazard ratio (95% CI)
Length of stay in rehabilitation (year)	–0.306	0.736 (0.495 to 1.096)	–0.180	0.835 (0.733 to 0.951)*	–0.171	0.842 (0.777 to 0.912)**
Age at PD diagnosis	0.037	1.038 (0.957 to 1.125)	0.080	1.084 (1.046 to 1.124)**	0.063	1.065 (1.039 to 1.091)**
**Sex**						
Female	Ref.		Ref.		Ref.	
Male	–0.426	0.653 (0.187 to 2.284)	0.910	2.484 (1.479 to 4.171)*	0.550	1.733 (1.281 to 1.281)*
**Charlson Comorbidity Index**						
0	Ref.		Ref.		Ref.	
1	1.408	4.086 (0.492 to 33.930)	0.279	1.322 (0.651 to 2.684)	1.087	2.964 (1.836 to 4.787)
> 2	0.717	2.048 (0.391 to 10.726)	0.504	1.655 (0.892 to 3.070)	1.132	3.101 (2.051 to 4.687)
**Health insurance type**						
Regional subscriber	Ref.		Ref.		Ref.	
Workplace subscriber	0.845	2.327 (0.612 to 8.844)	0.021	1.021 (0.575 to 1.815)	0.383	1.466 (1.014 to 2.121)*
Medical aid beneficiary	–0.808	0.446 (0.019 to 10.290)	0.365	1.441 (0.378 to 5.489)	0.342	1.408 (0.647 to 3.065)
**Region**						
Capital region	Ref.		Ref.		Ref.	
Non-capital region	–1.411	0.244 (0.070 to 0.845)*	–0.068	0.934 (0.579 to 1.508)	–0.175	0.839 (0.618 to 1.140)
**Insurance premium deciles**						
0∼2	Ref.		Ref.		Ref.	
3∼8	–1.448	0.235 (0.027 to 2.063)	–0.134	0.874 (0.360 to 2.125)	–0.002	0.998 (0.560 to 1.779)
9∼10	–0.368	0.692 (0.107 to 4.455)	–0.090	0.914 (0.385 to 2.170)	–0.171	0.843 (0.467 to 1.519)
LEDD***	0.254	1.289 (0.928 to 1.791)	–0.038	0.962 (0.845 to 1.096)	0.029	1.029 (0.955 to 1.109)
Total time in receipt of rehabilitation services***	0.245	1.278 (0.808 to 2.021)	–0.043	0.957 (0.809 to 1.133)	0.006	1.006 (0.900 to 1.125)

CI, confidence interval; Ref, reference group; SE, standard error. *p < 0.05; **p < 0.0001 ***LEDD and total time were log-transformed due to non-normal distribution.

## 4 Discussion

Although the positive effects of rehabilitation in patients with PD have previously been investigated, few studies have assessed its association with mortality using longitudinal national claims databases (Mak et al., 2017; Ypinga et al., 2018; Dommershuijsen et al., 2023). In addition, studies in which rehabilitation services have been investigated as a potential protective factor that could reduce mortality in patients with PD are insufficient (Dommershuijsen et al., 2023). Our study investigated the relationship between the length of stay in rehabilitation services (occupational and physical therapy) and mortality in patients with PD using national health insurance data. The results indicated that a longer duration of rehabilitation is associated with a tendency toward decreased mortality risks. These findings provide solid evidence supporting the importance of rehabilitation for individuals with PD.

Our findings provide evidence that rehabilitation services, including occupational and physical therapy, could be critical for patients with PD. Several studies have examined the effectiveness of rehabilitation for patients with PD (Frazzitta et al., 2014; Chen et al., 2021; Lo Buono et al., 2021). However, their evidence in support of the effectiveness of rehabilitation was very limited. Previous research has focused on the short-term and immediate effects of specific therapies (Abbruzzese et al., 2016; Davide et al., 2018; Chen et al., 2021; Lo Buono et al., 2021). While these studies do highlight the necessity of rehabilitation, they fail to examine the long-term effects and functional results. Our study supplemented the limitations of those studies by investigating the relationship between the length of rehabilitation and mortality using long-term follow-up results, achieved by taking advantage of national claims data.

Although our findings suggest that rehabilitation services may reduce the risk of mortality among patients with PD, the utilization of these services is low in Korea (Seo et al., 2018; Yun and Seo, 2021). Rehabilitation may enhance quality of life and reduce mortality risk by increasing the physical, cognitive, and emotional function of patients with PD (Choi and Cho, 2022; Yoon, 2022). In Korea, the number of patients with PD has been increasing over time; however, rehabilitation services for patients with PD have not increased to meet this need (Seo et al., 2018; Yun and Seo, 2021). According to Seo et al. (2018), more than 60% of patients with PD in Korea between 2004 and 2015 did not receive rehabilitation services (Seo et al., 2018). Thus, there is a lack of professional rehabilitation for patients with PD in Korea.

Conversely, rehabilitation services for patients with PD are frequently utilized in the Netherlands (Seo et al., 2018; Ypinga et al., 2018). Researchers and clinicians in the Netherlands introduced ParkinsonNet to provide evidence-based therapy through specialized therapist training (Nijkrake et al., 2010). The outcomes of ParkinsonNet, interventions by these specialized therapists were found to be more cost-effective than the usual interventions by therapists who had not received this training. The service was shown to reduce PD-related complications, improve daily functioning, and contribute to the quality of life of PD caregivers (Bloem et al., 2017; Ypinga et al., 2018). In Korea, rehabilitation is primarily targeted to patients with conditions like stroke, dementia, mild cognitive impairment, and spinal cord injuries (Kim et al., 2020). However, the growing number of patients with PD means there is a need to promote rehabilitation specifically for this patient demographic. It is crucial to invest effort into the development of innovative treatment models and guidelines, such as the ParkinsonNet initiative in the Netherlands, tailored to patients with PD. This will provide cost-effective management methods to PD patients and their caregivers, mirroring the positive effects of ParkinsonNet.

Multidisciplinary approaches that combine pharmacotherapy and rehabilitation can improve the quality of life of patients with PD. Previous studies suggest that multidisciplinary approaches more effectively improve the physical function and quality of life of patients with PD (Lo Buono et al., 2021; Mitsui et al., 2021; Steendam-Oldekamp et al., 2023). Steendam-Oldekamp et al. (2023) study reported that those who received multidisciplinary programs had improved executive function and delayed admission to nursing homes compared to a control group (Steendam-Oldekamp et al., 2023). Lo Buono et al. (2021) found that multidisciplinary approaches led to increased physical and cognitive function, mood, and independence in activities of daily living function. Our findings support these, by demonstrating a positive association between the length of rehabilitation and mortality. Our results further indicate that the mortality rate may be lower for those who start rehabilitation sooner, even when controlling for LEDD. The addition of rehabilitation to pharmacotherapy can be beneficial to patients with PD.

Previous studies have explored the factors associated with the mortality of patients with PD (Willis et al., 2012; Zhang et al., 2022). These factors can be categorized as non-modifiable and modifiable (Willis et al., 2012). To reduce mortality risks, health professionals should prioritize interventions targeting modifiable rather than non-modifiable factors. Non-modifiable factors include such as sex, ethnicity, and genetic inheritance. Modifiable factors include drinking, eating habits, and mental health. These latter have been extensively investigated as potential targets for the reduction of mortality (Paul et al., 2019; Zhang et al., 2022). Our study provides evidence that rehabilitation may be among the modifiable factors able to reduce mortality rates in patients with PD.

Our results need to be compared with those of similar studies of different ethnicities and conducted in other countries. Previous studies have revealed the positive effects of rehabilitation services on functional status and have shown an association between physical activity (Frazzitta et al., 2012; Welsby et al., 2019; Doucet et al., 2021; Elena et al., 2021; El Hayek et al., 2023), a component of rehabilitation services, and mortality (Zhang et al., 2022). However, to our knowledge, this is the first study to examine the direct association between rehabilitation services and mortality. Furthermore, the literature points to significant ethnic differences in mortality among PD patients (Fernandes et al., 2015; Ben-Joseph et al., 2020). Yet, we could not determine if there is a difference in the association between rehabilitation services and mortality among various ethnic groups or countries. These comparisons should be made in future research.

Our findings revealed no significant association between the duration of rehabilitation and all-cause mortality among patients who received only occupational therapy. In contrast, significant relationships were observed in patients who underwent physical therapy and those who received both occupational and physical therapy. These outcomes may be attributable to the small sample size, as indicated by the larger standard error in the results comparing the duration of rehabilitation with all-cause mortality (Suresh and Chandrashekara, 2012; Thiese et al., 2016; Gómez-de-Mariscal et al., 2021). The *p*-values, which are influenced by sample size, suggest that larger samples may reveal significant relationships if they exist (Suresh and Chandrashekara, 2012; Thiese et al., 2016; Gómez-de-Mariscal et al., 2021). In our study, only 39 patients with PD received occupational therapy exclusively. Furthermore, the standard error for the relationship between the duration of rehabilitation and all-cause mortality was larger for this group than the others. Consequently, future studies should recruit more patients with PD who have undergone occupational therapy to compare their all-cause mortality rates with those receiving other therapies.

### 4.1 Study limitations

Our study had several limitations. First, we could not control for all covariates that might be associated with mortality, such as physical or cognitive function, because the Korean National Health Insurance Service data primarily includes information relevant to insurance rather than functional status. Despite this limitation, we incorporated LEDD into our analysis as a measure of the severity of PD symptoms. Second, rehabilitation typically comprises physical, occupational, and speech therapy, but speech therapy is not covered by insurance services in Korea. As a result, we could not include speech therapy data in our outcome measure. Third, the rehabilitation code in Korea is not divided into inpatient and outpatient. Thus, we could not distinguish between those patients who received rehabilitation services in inpatient or outpatient settings. This, again, is likely to reflect severity.

## Data availability statement

The datasets utilized in this research are the property of the Korea National Health Insurance Service (NHIS) and access is granted following approval via a designated procedure. Requests to access these datasets should be directed to Korea National Health Insurance Service (NHIS).

## Ethics statement

The data used in this study was approved by the Korea National Health Insurance Service Inquiry Commission and the Institutional Review Board (IRB) of Wonju Severance Christian Hospital (IRB No. CR321308). In addition, this study has been reviewed and approved by the IRB of the Yonsei University Mirae Campus (IRB No. 1041849-202307-SB-128-01). Written informed consent for participation was not required from the participants or the participants’ legal guardians/next of kin in accordance with the national legislation and institutional requirements.

## Author contributions

SB: Data curation, Formal analysis, Investigation, Writing–original draft. IH: Conceptualization, Formal analysis, Investigation, Project administration, Writing–review and editing. MB: Funding acquisition, Project administration, Writing–review and editing.
