# Supplementary material for: Association between the length of stay in rehabilitation and mortality among the adults with Parkinson’s disease: 2009–2019 Korean National Health Insurance Service Databases
Source: Front Aging Neurosci. 2024 Aug 5;16:1428972. doi: 10.3389/fnagi.2024.1428972 (PMC11330883; doi:10.3389/fnagi.2024.1428972)
Supplement: Supplementary file 1 [file Table_1.DOCX]

Supplemental Material

Table S1. Description of codes for rehabilitation services in south Korea

| Code | Description |
| --- | --- |
| Occupational therapy |  |
| MM111. Simple occupational therapy | This is calculated when an occupational therapist conducts training for 10 minutes or more at the same time for two or more patients. |
| MM112. Complex occupational therapy | This is calculated when an occupational therapist conducts focused one-on-one therapy for 10 minutes to approximately 30 minutes. |
| MM113. Special occupational therapy | This is calculated when an occupational therapist conducts focused one-on-one therapy for more than 30 minutes, conducting various therapies. |
| MM114. Activities of Daily Living Training | This is calculated when an occupational therapist conducts focused one-on-one training for at least 20 minutes on adapting to daily life activities such as eating, dressing and undressing, toileting, and hygiene training. |
| Physical therapy |  |
| MM301. Mattress or Mobilization Training | This is calculated for individuals with central nervous system disorders such as hemiplegia, paraplegia, quadriplegia, cerebral palsy, or those with limb amputations, etc., who have limitations in movement actions, when mattress training, mobilization training, incline training, and wheelchair training, etc., are conducted for more than 30 minutes. |
| MM302. Gait Training | This is calculated for individuals with central nervous system disorders such as hemiplegia, paraplegia, quadriplegia, cerebral palsy, or those with limb amputations, etc., who have limitations in walking actions, when gait training is conducted for more than 30 minutes. |
| MM105. Rehabilitative Development Therapy for Disorder of Central Nervous System | This is calculated when a rehabilitation medicine specialist or physical therapist, who has completed more than 120 hours of training courses such as Vojta or Bobath techniques, conducts focused one-on-one therapy for more than 30 minutes for the treatment of developmental delays and muscle paralysis and stiffness due to central nervous system disorders. |
